# Sex differences in coronary artery disease

**DOI:** 10.1007/s12471-021-01619-x

**Published:** 2021-09-10

**Authors:** J. H. Kuneman, J. J. Bax

**Affiliations:** 1 Department of Cardiology, Leiden University Medical Center, Leiden, The Netherlands; 2 Turku Heart Centre, University of Turku and Turku University Hospital, Turku, Finland

Cardiovascular disease, predominantly ischaemic heart disease, remains the leading cause of disease burden in men and women worldwide [1]. Over the past decades, considerable evidence has been reported on sex-related differences, in terms of prevalence and severity of coronary artery disease (CAD) and outcomes. Among patients with suspected acute or chronic coronary syndrome, women present more frequently, and at older age, with different chest pain symptoms than men, and they have higher cardiovascular risk profiles but less obstructive CAD [2, 3]. Accordingly, concerns have been raised that women are likely to receive suboptimal treatment for CAD compared with men.

Coronary computed tomography angiography (CCTA) is an accurate diagnostic test for symptomatic patients with suspected CAD [4]. It enables comprehensive, noninvasive evaluation of CAD, including coronary anatomy and luminal narrowing, and quantification of atherosclerosis using advanced plaque analysis. A previous CCTA study demonstrated less calcified plaques and more noncalcified plaques in women than in men [5]. Moreover, it has been noted that women have less coronary plaque burden on a per-patient level than men (with lower fibrous/fibrofatty plaque volume and total plaque volume) [6]. In addition, women show significantly slower progression of coronary atherosclerosis on serial CCTA scans [7]. Yet, women with ischaemic heart disease have worse outcomes compared with men [8].

In this issue, Arslan et al. report on sex-related differences in the clinical presentation and CCTA results and their effect on downstream outpatient testing among patients presenting with suspected acute coronary syndrome (ACS) to the emergency department [9]. This subanalysis of the Better Evaluation of Acute Chest Pain with Coronary Computed Tomography Angiography (BEACON) trial, in which CCTA was evaluated against standard of care [10], included 500 patients, of whom 47% were women. Study endpoints were recorded within 30 days after the initial presentation of suspected ACS and included coronary angiography, coronary revascularisation, hospital admission, length of stay, repeated emergency department visits and subsequent outpatient testing. The authors reported that women had a lower incidence of obstructive CAD on CCTA compared with men, despite being older (56 ± 10 vs 53 ± 10 years, *p* < 0.01) and less frequently using statins (18% vs 28%, *p* = 0.01). In addition, women with suspected ACS were less frequently admitted to hospital and had less outpatient testing if CCTA was used in the diagnostic work-up.

The intriguing data presented by Arslan et al. are in line with current evidence and emphasise the apparent sex differences among patients presenting with suspected ischaemic heart disease. Numerous reasons exist to explain the sex disparities in CAD, including, but not limited to, potential differences in pathophysiology as well as (patients’ and physicians’) disease perception. In general, the lower prevalence of obstructive CAD in women versus men can be a reassuring sign for the treating physician. Historically, the presence of less obstructive CAD in women has been associated with lower referral rates for invasive coronary angiography and a shift towards alternative extra-cardiac diagnoses [11]. In the study [9], women had more non-obstructive CAD. Of note, 48% of the women who presented with suspected ACS at the emergency department had no coronary artery stenosis on CCTA (compared with 39% of men), which may be an explanation for less downstream outpatient testing in the female patients.

Although data from a large study showed that patients with non-obstructive CAD fared much better than those with obstructive CAD [11], the presence of non-obstructive CAD in women with anginal symptoms may not necessarily be a reassuring sign. The Women’s Ischemia Syndrome Evaluation (WISE) study reported that women with cardiac symptoms and no or minimal stenoses on invasive coronary angiography have a 2-fold and 4‑fold increased risk of adverse cardiovascular events, respectively, compared with asymptomatic women [12]. These observations, along with similar findings from other studies, may suggest a sex-specific pathophysiology for ischaemic heart disease.

It has been hypothesised that dysfunction of the coronary microvasculature and endothelium contributes to the worse prognosis of symptomatic women in the absence of obstructive CAD [2]. Impaired regulation of myocardial blood flow can be assessed by coronary flow reserve (CFR) on myocardial perfusion imaging. Reduced CFR may indicate microvascular or endothelial dysfunction in the absence of epicardial coronary stenosis [13]. In a study evaluating symptomatic patients who were referred for invasive coronary angiography, women with severely impaired CFR had a significantly increased risk of adverse cardiovascular events compared with men (hazard ratio 2.49, 95% confidence interval 1.16–5.38) [14].

Arslan et al. observed that the use of CCTA in the work-up of patients with suspected ACS is feasible and is associated with less downstream outpatient testing in women [9]. However, whether this affects clinical outcomes or contributes to the underdiagnosis of ischaemic heart disease in women remains unanswered. Long-term follow-up data may provide further insight into these sex differences in CAD.
